# The prognostic significance of high/positive expression of tissue VEGF in ovarian cancer

**DOI:** 10.18632/oncotarget.25702

**Published:** 2018-07-17

**Authors:** Bing-Qin Guo, Wen-Qiao Lu

**Affiliations:** ^1^ Department of Pathology, The First Affiliated Hospital of Bengbu Medical College, Anhui 233003, China; ^2^ The Interventional Diagnosis and Treatment Ward, Thoracic Hospital in Shandong Province, Shandong 250013, China

**Keywords:** VEGF, ovarian cancer, prognosis

## Abstract

**Background & aim:**

At present, numerous reports have shown that high/positive expression of tissue vascular endothelial growth factor (VEGF) may be associated with the prognosis of patients with ovarian cancer. However, their results still remained controversy. Thus, this meta-analysis was designed to analyze and assess the prognostic value of tissue VEGF expression in patients with ovarian cancer.

**Method:**

We searched PubMed, Embase, Cochrane Library and Web of Science to October, 2017. Hazard Ratio (HR) with its 95% confidence intervals (CIs) was used to evaluate the association between high/positive expression of tissue VEGF and the prognosis of ovarian cancer patients. All statistical analyses were performed using standard statistical procedures provided in RevMan 5.2.

**Result:**

A total of 18 studies (including 1145 patients) were included for this meta-analysis. The positive/high expression of tissue VEGF had an obvious association with overall survival (OS) (HR 2.24, 95% CI 1.36–3.70; *P*=0.002), progression-free survival (PFS) (HR 1.60, 95% CI 1.11–2.31; *P*=0.01) and disease-free survival (DFS) (HR 3.49, 95% CI 1.27–9.56; *P*=0.02) of patients with ovarian cancer respectively.

**Conclusion:**

The present meta-analysis indicated that positive/high expression of tissue VEGF may have a close association with survival of ovarian cancer.

## INTRODUCTION

Cancer, especially ovarian cancer in women, is a major public health problem worldwide. Ovarian cancer is currently the fifth leading cause of cancer deaths in women, associated with an estimated 14,180 deaths in the United States in 2015 [1]. According to the report, the estimated new cases of ovarian cancer is 21,290 and locates in the third place in genital system cancer, which only rank next to the incidence of uterine cervix and corpus cancer. Despite surgical resection is the optimal option, patients with ovarian cancer usually experience poor prognosis, possibly due to a lack of diagnosis in early stage. The majority of patients are diagnosed with advanced disease, and at least nearly half of patients dead within 5 years after their diagnosis [1]. Thus, appropriate prognostic markers are needed to predict patients’ post-treatment prognosis and survival of patients at high risk of recurrence or metastasis.

At present, many advances have been made in the treatment approaches, imaging techniques and staging procedures. However, there is no effective method indicating the prognosis of patients before operation or treatment, for the Federation International of Gynecology and Obstetrics (FIGO) stage which is usually accepted to indicate the prognosis of gynecological tumors must be made after surgery according to a precise pathologic result. For decades, many studies have shown that various growth factors could stimulate angiogenesis in physiological and pathological conditions. Among these, vascular endothelial growth factor (VEGF) has been shown to play a major role in the proliferation and migration of endothelial cells, providing nourishment to the growing tumors and allowing the tumor cells to establish continuity with the host vasculature [2]. Recent studies have demonstrated a significant correlation between VEGF expression and microvessel density (MVD) in malignant tumors arising from several organs [3]. In addition, several studies have demonstrated that serum VEGF has a significant association with the prognosis of ovarian cancer [4–7]. In recent years, numerous reports have shown that high/positive expression of tissue VEGF may be associated with the prognosis of patients with ovarian cancer [8–25]. However, their results still remain controversy. Thus, this meta-analysis is designed to analyze and assess the prognostic value of tissue VEGF expression in patients with ovarian cancer.

## RESULTS

### Included studies and characteristics

After five studies are excluded (two studies for lack of available data, two studies are about cervical cancer, one study for just review), eventually a total of 18 studies (including 1145 patients) are included in this meta-analysis, of which 12 studies are included to analyze the association between positive/high expression of tissue VEGF and OS of patients with ovarian cancer. Nine and five studies are included to analyze the association between positive/high expression of tissue VEGF and PFS and DFS of patients with ovarian cancer respectively. Of the included studies, six studies detect the expression of tissue VEGF with polymerase chain reaction (PCR), 11 studies detect the expression of tissue VEGF with immunohistochemistry (IHC). Seven studies were conducted in Japan, three in Italy, three in USA, one in Germany, one in Austria, one in China, one in UK and one in Egypt. The sample sizes ranged from 18 to 97 patients (Table 1).

**Table 1:** The characteristics of included studies for the present meta-analysis

Study	Country	N	Median age (year)	Cut-off value	FIGO stage	Study design	Detection method	Follow-up time (month)	Outcome	n	Quality score
Brustmann H. 2004	Austria	41	NR	10%	I-III	NR	IHC	22	DFS	21	19
Gadducci A et al. 2003	Italy	45	NR	≥ 4+	NR	Retrospective	IHC	NR	OS, PFS	14	15
Garzetti GG et al. 1999	Italy	32	58.5 (34-69)	NR	I-III	Retrospective	IHC	37 (12-96)	DFS	15	17
Goodheart MJ et al. 2005	USA	77	50 (21-85)	≥ 4+	I	Retrospective	IHC	73 (24-176)	PFS	5	18
Hartenbach EM et al. 1997	USA	18	66.5 (29-83)	25 cycles	III/IV	NR	RT-PCR	49.5 (15-78)	OS	12	16
Hata K et al. 2004	Japan	85	54 (19-81)	0.14	I-IV	Retrospective	PCR	NR	PFS	43	19
Ino K et al. 2006	Japan	67	NR	50%	I-IV	Retrospective	IHC	60 (1-121)	OS, PFS	49	19
Li L et al. 2009	China	78	(34-72)	10%	I-IV	NR	IHC	NR	OS, DFS	46	15
Nakayama K et al. 2002	Japan	60	53 (22-81)	Positive	I-IV	Retrospective	PCR	40.6 (6-128)	OS, PFS	30	15
Nishida N et al. 2004	Japan	80	54.4 (23-79)	10%	I-IV	Retrospective	IHC	33.2 (2.75-89.75)	DFS	58	17
Raspollini MR et al. 2004	Italy	83	60 (33-79)	30%	III	Retrospective	IHC	44.8 (3-204)	OS, DFS	50	19
Secord AA et al. 2007	USA	67	NR	>1.2 fold to actin	NR	Retrospective	Immunoblot	NR	OS, PFS	33	17
Shen GH et al. 2000	Japan	64	54 (21-88)	25%	I-IV	Retrospective	IHC	31 (3-120)	OS	31	19
Shimogai R et al. 2008	Japan	66	55.4 (21-78)	4%	I-IV	Prospective	RT-PCR	154 (92.9-398.5)	OS, PFS	26	19
Sinn BV et al. 2009	Germany	97	58 (34-80)	mRNA: 30.52	I-IV	NR	RT-PCR	38 (2-83)	OS, PFS	NR	15
Sundar SS et al. 2006	UK	88	63 (25-88)	NR	I-IV	Retrospective	IHC	28.5 (0.49-165.3)	OS, PFS	NR	19
Ueda M et al. 2005	Japan	73	NR	50%	I-IV	NR	IHC	NR	OS	36	15
Kassim SK et al. 2004	Egypt	24	43.5 (21-65)	120pg/mg	I-IV	Retrospective	RT-PCR	36	OS	NR	17

Abbreviations: N, total number of eligible patients; n, number of patients with high/positive tissue VEGF; OS, overall survival; DFS, disease-free survival; PFS, progression-free survival; NR, not report; RT-PCR, reverse-transcriptase polymerase chain reaction; IHC, immunohistochemistry.

**Figure 1:**
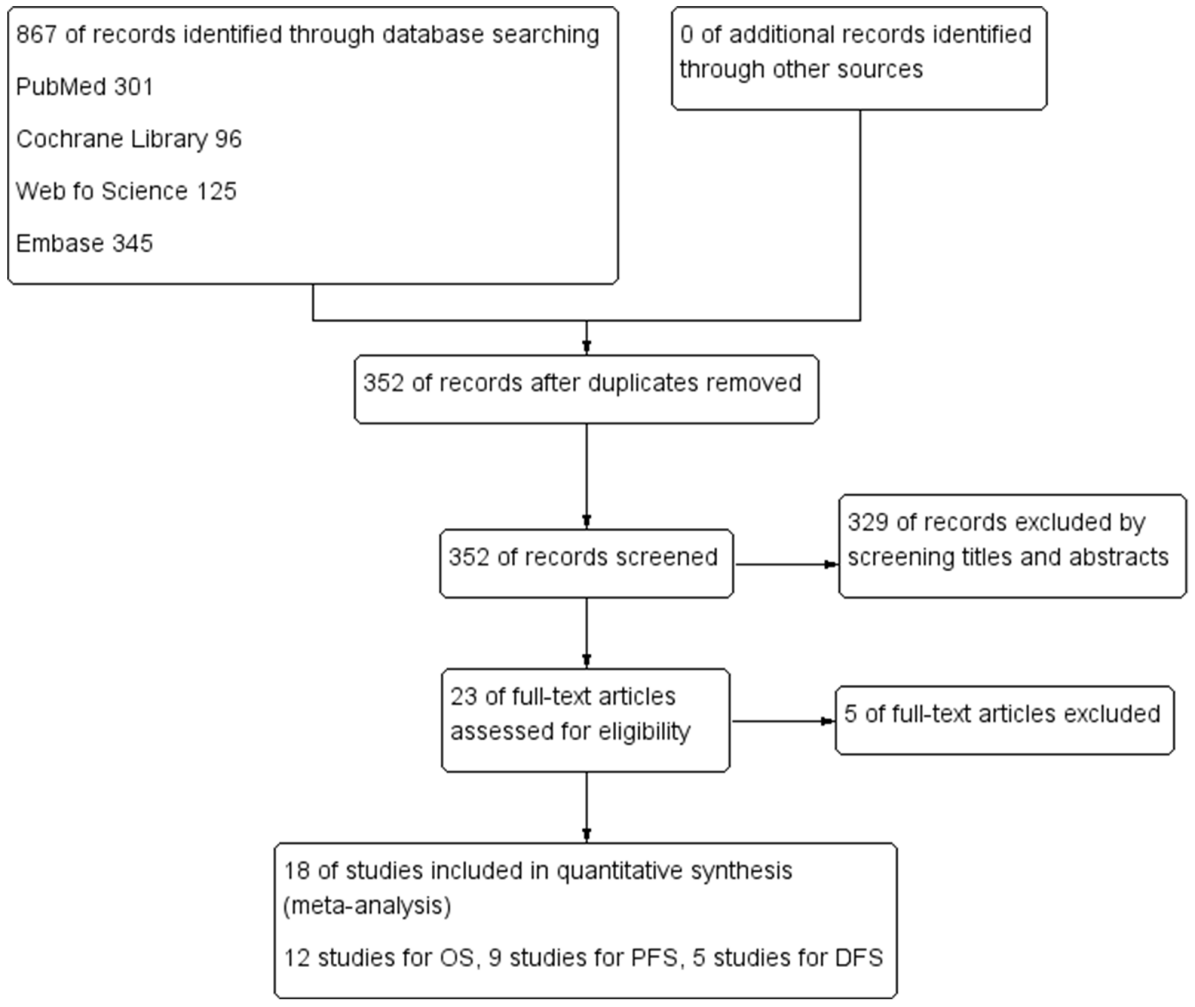
Flow diagram following the PRISMA template of the search strategy for studies included in this meta-analysis

The detail search process and summary of studies were showed in study flow diagram (Figure 1). The other study characteristics were showed in Table 1.

### The association between high/positive expression of tissue VEGF and OS of ovarian cancer patients

**Figure 2:**
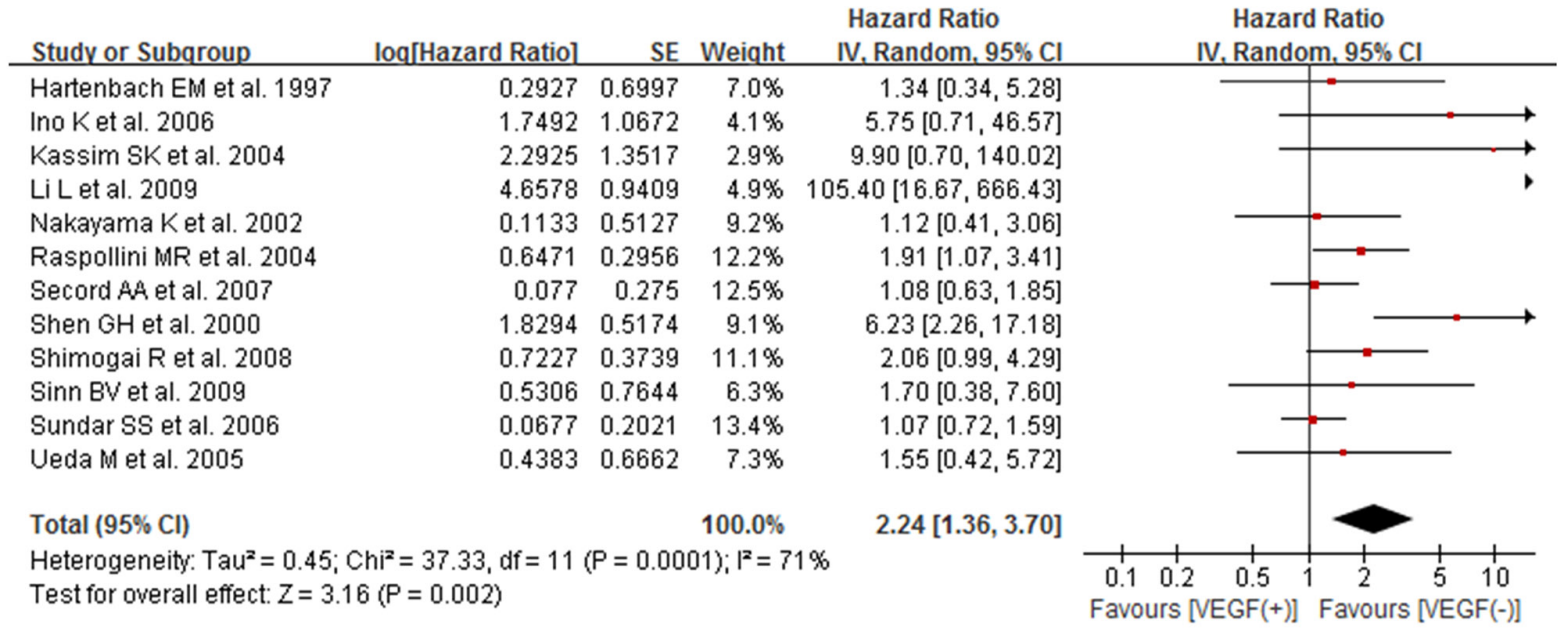
Forest plot for the association between high/positive expression of tissue VEGF and OS of ovarian cancer patients

A total of 12 studies [12, 14–17, 20–25] were included to analyze the relationship of positive/high tissue VEGF expression with overall survival (OS) in patients with ovarian cancer. Pooled result of meta-analysis showed significant correlation between positive/high expression of tissue VEGF and overall survival, with the pooled HR being 2.24 (95% CI 1.36–3.70; *P*=0.002). As much significant heterogeneity between studies (*I*^*2*^=71% and *p*=0.0001), a random-effect model was used (Figure 2).

### The association between high/positive expression of tissue VEGF and PFS of ovarian cancer patients

**Figure 3:**
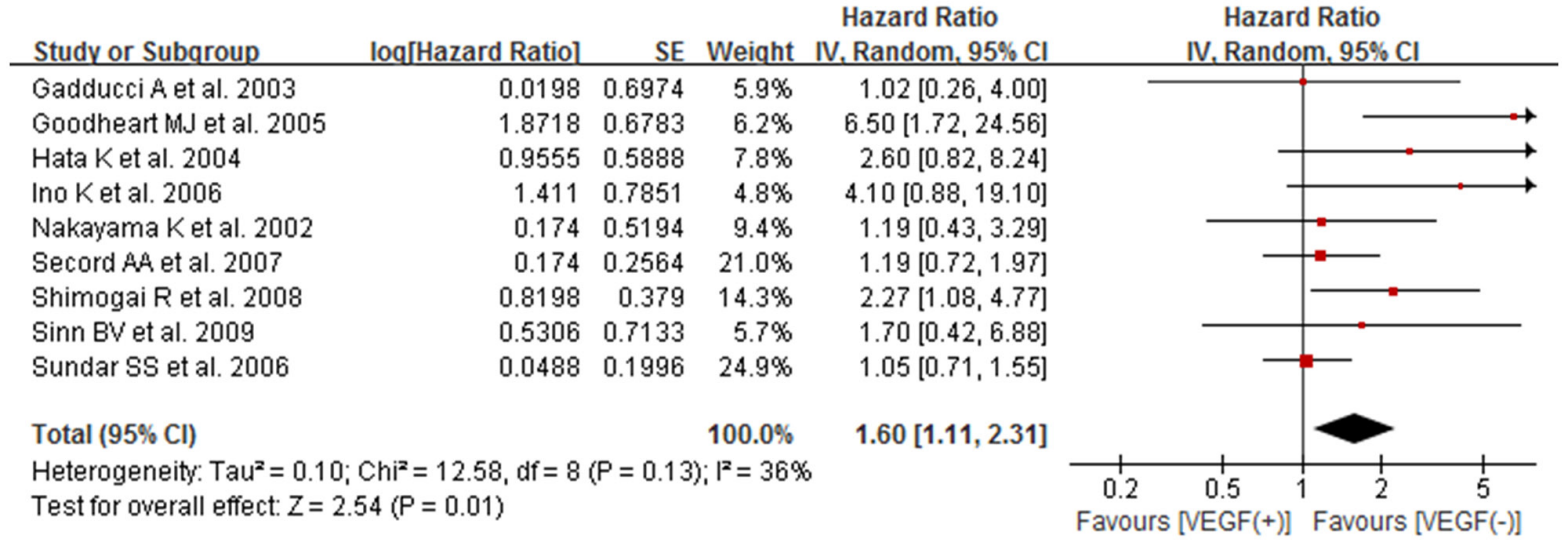
Forest plot for the association between high/positive expression of tissue VEGF and PFS of ovarian cancer patients

Nine studies reported the association between positive/high expression of tissue VEGF and progression-free survival (PFS) [9, 11, 13, 14, 17, 20, 22–24]. We also used random-effect model to estimate the pooled result. The combined result indicated that the expression of tissue VEGF had significant association with PFS of patients with ovarian cancer (HR 1.60, 95% CI 1.11–2.31; *P*=0.01) (Figure 3).

### The association between high/positive expression of tissue VEGF and DFS of ovarian cancer patients

**Figure 4:**
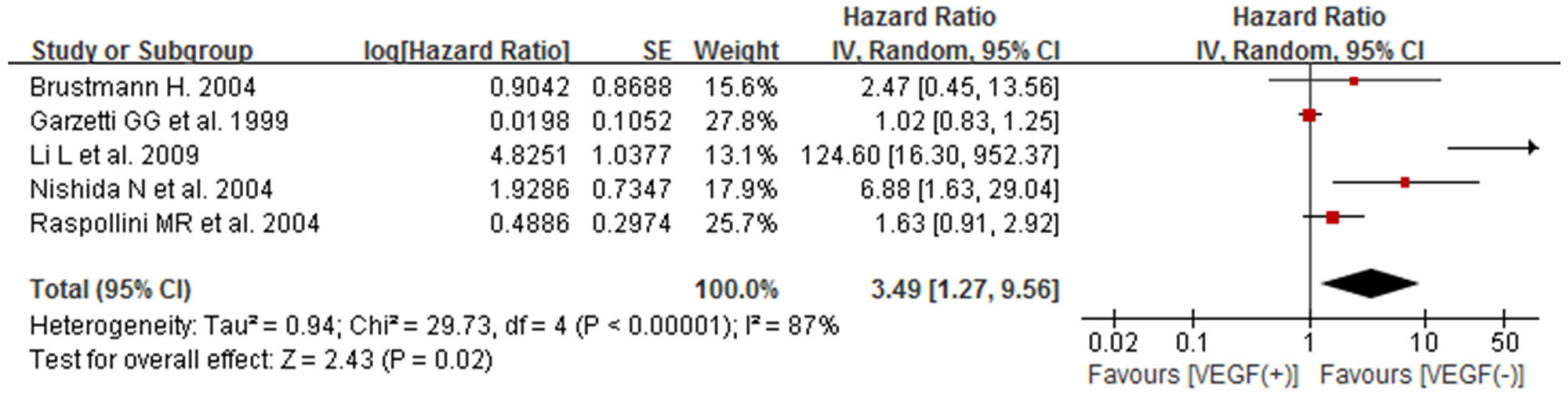
Forest plot for the association between high/positive expression of tissue VEGF and DFS of ovarian cancer patients

Considering significant heterogeneity between studies (*I*^*2*^=87% and *p*<0.00001), a random-effect model was used to estimate the association between tissue VEGF expression and DFS of patients with ovarian cancer. The pooled HR indicated that positive/high expression of tissue VEGF had an obvious association with DFS in patients with ovarian cancer (HR 3.49, 95% CI 1.27–9.56;*P*=0.02) (Figure 4).

### Subgroup and publication bias

In order to analyze the prognostic effects of positive/high tissue VEGF expression on OS, we conducted subgroup analysis grouping according to detection method of tissue VEGF expression (IHC or PCR) and quality score (15, 16–18, 19) of included studies. All pooled results were estimated with fixed-effect model because of good homogeneity among the studies, except subgroup of IHC. Statistically significant effect of tissue VEGF expression on OS was observed in all subgroups except quality score of 16–18. For the subgroups according to detection method, significant results were found in subgroups of both IHC (HR 3.70; 95% CI 1.44, 9.52; *P*=0.007) and PCR (HR 1.73; 95% CI 1.05, 2.86; *P*=0.03). For the subgroups according to quality score, significant results were also found in subgroups of both score=15 (HR 2.35; 95% CI 1.22, 4.53; *P*=0.01) and score=19 (HR 2.17; 95% CI 1.18, 3.98; *P*=0.01) (Table 2).

**Table 2:** The pooled results of subgroups for the association between high/positive expression of tissue VEGF and OS

Subgroups	Pooled results		P value	Analytical effect model
	HR	95% CI		
Detection method				
IHC	3.70	1.44, 9.52	0.007	Random-effect model
PCR	1.73	1.05, 2.86	0.03	Fixed-effect model
Quality score				
15	2.35	1.22, 4.53	0.01	Fixed-effect model
16-18	1.11	0.67, 1.84	0.68	Fixed-effect model
19	2.17	1.18, 3.98	0.01	Fixed-effect model

Abbreviations: HR, hazard ratio; CI, confidence intervals.

**Figure 5:**
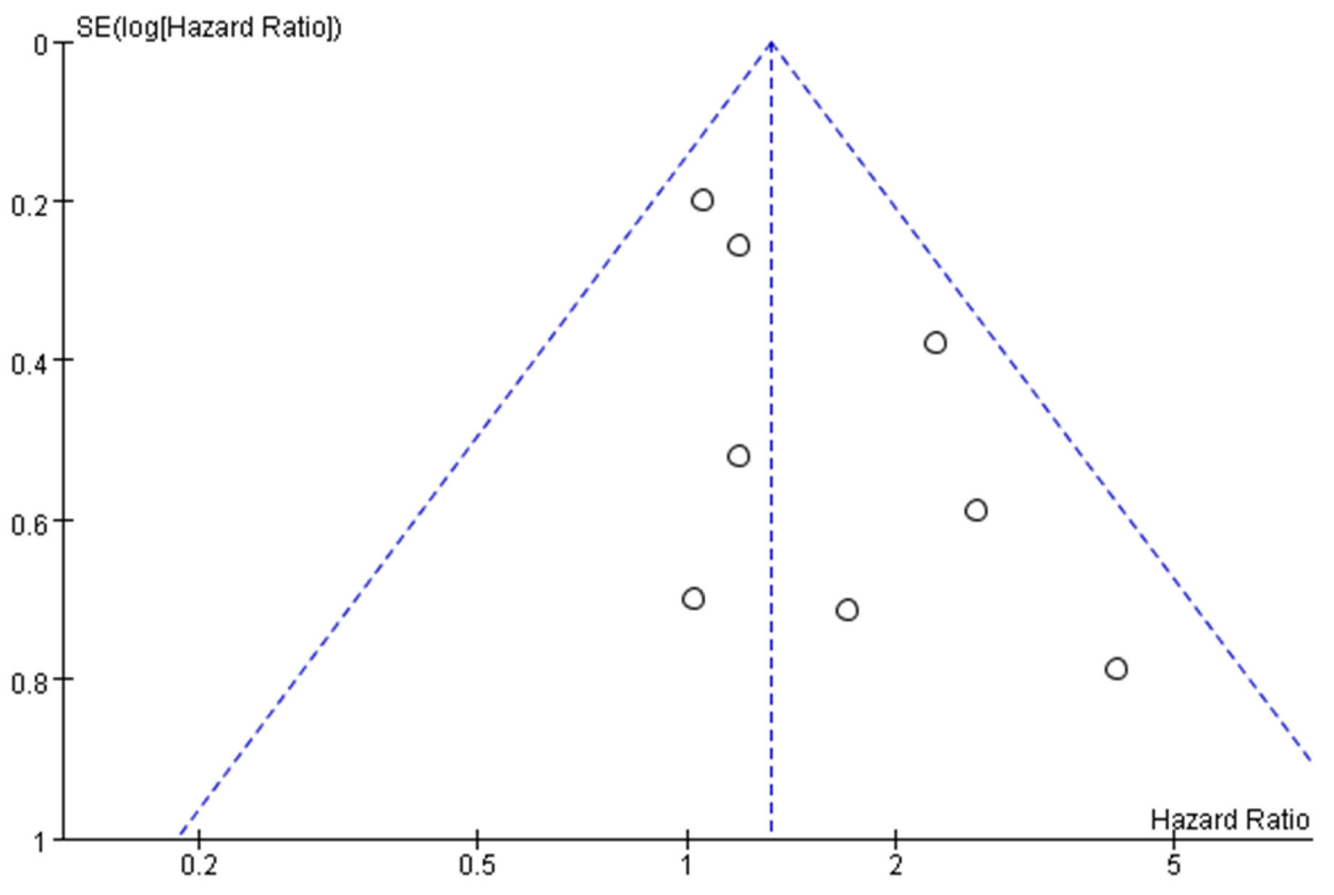
Funnel plots for detecting publication bias of the included studies

Funnel plots were conducted for assessing the publication bias of included literatures and we could roughly assess the publication bias by seeing whether their shapes were of any obvious asymmetry. The funnel plots showed no obvious evidence of publication bias with regard to the effects on OS (Figure 5).

## DISCUSSION

Recently, it was given increasing interest that high/positive expression of tissue VEGF may be associated with the prognosis of patients with ovarian cancer. VEGF is a heparin binding dimeric glycoprotein, which is related with angiogenic, mitotic, and microvascular permeability-inducing activities, leading to the extravasation of plasmaproteins and to proangiogenic stromal changes [26]. It has been earlier implicated that various paracrine effects of VEGF are always mediated by binding with high affinity to the tyrosine kinase receptors flt-1 and KDR/flk-1 [27]. Previous animal experimental study revealed the relevance of VEGF in tumor tissue by blockage of VEGF-induced stimulation endothelial cells *in vitro*, which prevented VEGF dependent tumor vascularization, growth, and metastases of mouse ovarian tumor cells in syngeneic mice [28]. Previously, several studies investigated VEGF expression in serous carcinomas in relation to proliferation [27, 29, 30]. In addition, several studies have demonstrated that both serum and tissue VEGF have a significant relevance with the prognosis of ovarian cancer [4, 6, 12, 13, 18]. To our best knowledge, Brustmann H initially reported a reciprocal relation of VEGF in serous ovarian carcinomas in 2004 [8]. It was found that the expression of VEGF seemed to be relevant to outcome, tumor stage, and grade, which may be helpful in understanding growth and spread of serous ovarian carcinomas [8]. Garzetti GG et al. in 1999 suggested that VEGF expression contributed to neoplastic invasiveness probably related to 72-kilodalton metalloproteinase [10]. They found that in cystoadenocarcinomas, expression of VEGF is related to the MMP2 index, suggesting that VEGF has a role in neoplastic invasiveness. It has been suggested that VEGF family members (VEGF-A, VEGF-B, VEGF-C, VEGF-D), basic fibroblast growth factor, and thymidine phosphorylase are expressed in a variety of human tumors in different ways [31–35]. VEGF-A, also known as vascular permeability factor, is considered to play an important role in tumor angiogenesis [35]. It has been shown that VEGF-C expression is associated with hyperplasia of lymphatic vessels [34]. It is conceivable that VEGF-C might play a crucial role in lymphatic proliferation and also in spread of solid tumors. Ueda M et al. reported that VEGF-C may play a critical role for the prevalent progression of the tumor by inducing tumor invasion, lymph node metastasis, and vascularity and subsequently inhibiting apoptosis in ovarian carcinomas [25]. In the present study, we included all of VEGF family members expressing in tumor tissue.

Though many studies reported the significant association between high or positive expression of VEGF and prognosis of ovarian cancer, their results still remained controversy. The purpose of this study was to investigate and analyze the high/ positive expression of VEGF as a prognostic biomarker in ovarian cancer. We perform this meta-analysis with 18 studies including 1145 patients. Our analysis results statistically support the conclusions that high/ positive expression of VEGF had a significant association with the prognosis of ovarian cancer, with the pooled HRs were 2.24 (95% CI 1.36–3.70; *P*=0.002) in overall survival, 1.60 (95% CI 1.11–2.31; *P*=0.01) in progression-free survival and 3.49 (95% CI 1.27–9.56; *P*=0.02) in disease-free survival. In addition, we also conducted subgroup analysis to estimate the prognostic value of VEGF in different groups. Our analysis results showed that high/ positive expression of VEGF had a significant association estimating with fixed-effect model in all subgroups except the subgroup of quality score 16-18.

Several limitations, however, exists in this meta-analysis. One of the main limitations is the inconsistency of the cut-off values of VEGF (definition of positive/high expression of VEGF), which may directly affect the results of studies and result in between-study heterogeneity. The positive criteria of VEGF was various in these studies, as shown in Table 1. In the study of Kassim SK et al. 2004, they determined the cut-off for VEGF that maximizes the sum of sensitivity (5/6, 83.3%) and specificity (15/18, 83.3%) in discriminating good from poor outcome of the disease, with cut-off value of VEGF equal to 120 pg/mg protein. At this cutoff, there was a significant correlation between VEGF positivity and the poor survival of the patients (*X*^*2*^ = 9.0, *P* < 0.01). Western blot analysis showed that the subtype VEGF_165_ is the only VEGF subtype that has been detected in all the ovarian tumors included in the study [15]. The second limitation is the different clinical stages of ovarian patients. As is well known, the survival of tumor patients is significant associated with their clinical stages. In this study, the majority of included studies involved ovarian cancer patients with FIGO stage of I-IV and failed to discuss the prognostic significance of VEGF with different stages. More important, the clinical stage of patients seems related with the level of VEGF in tissue. In the study of Kassim SK et al, all stages of ovarian cancer patients showed significantly increased mean rank of VEGF over that in the benign group (mean ranks are 6.6, 15.5, 20.33, 25, and 30 in benign, stages I, II, III, and IV, respectively, *X*^*2*^ = 22.8, *P* < 0.001). Thirdly, all the studies we included were observational retrospective studies, expect studies that did no report their study design type. There is no prospective study in the analysis, which may affect the strength of evidence of analysis results. Finally, the prognosis of patients may also be influenced by other multiple factors, such as age of patients, therapy methods they received, histological type and VEGF detection methods, which should also be taken into consideration. Thus, further prospective researches need to be designed to clear the association between positive/high expression of tissue VEGF and prognosis of ovarian cancer patients with different clinical stages.

In conclusion, positive/high expression of tissue VEGF may have a close association with worse survival in patients with ovarian cancer considerating of the evident statistical significance. Elevated tissue VEGF expression may be used as a prognostic biomarker for early identification of poor prognosis.

## MATERIALS AND METHODS

### Including and excluding criteria of this meta-analysis

Including criteria: (1) All of randomized, controlled trials (RCTs), observational prospective or retrospective studies were included; (2) included people with a pathological diagnosis of ovarian cancer; (3) tumor tissue VEGF was detected by Immumohistochemical staining or polymerase chain reaction; (4) definition of positive/high expression of tissue VEGF was reported; (5) the outcomes including overall survival, disease-free survival, progression-free survival were reported; (6) sufficient data were provided.

Excluding criteria: (1) Trials on animals; (2) abstracts, letters, editorials, expert opinions, reviews, case reports; (3) patients having other primary tumors; (4) studies without sufficient data; (5) the definition of positive/high expression of tissue VEGF (cut-off value) did not give or meet our including criteria.

### Search strategy

We searched PubMed, EMBASE, Cochrane Library and Web of Science to October, 2017. Our searching terms and procedures were as follows: (1) “VEGF”; (2) “ovarian cancer” OR “ovarian carcinoma” OR “ovarian tumor”; (3) “prognosis” OR “survival” OR “outcome”. Search strategy was (1) AND (2) AND (3). Other related terms, including references of some literatures we read, were also searched in English. Two assessors independently screened the titles and abstracts of each study. Once relevant studies became certain, the full texts were obtained for further evaluation.

### Quality assessment

Two reviewers assessed the quality of all the included studies using REporting recommendations for tumor MARKer prognostic studies (REMARK) [36] independently, and the total scores of each study were displayed in the characteristics table (Table 1). The scores were judged according to relevant information about the study design, pre-planned hypotheses, patient and specimen characteristics, assay methods, and statistical analysis methods as well helpful presentations of data and important elements to include in discussions [36].

### Data extraction

Data for the analysis were extracted independently by two reviewers, and disagreement was resolved by their discussion. In addition, the extracted contents including total number of eligible patients, published years, country, number of patients with high/positive tissue VEGF, Median age, follow-up time, definition of positive/high expression or cut-off value, FIGO stage, study design, detection method, outcome, and quality score were extracted using a standardized form.

Data collected were input into RevMan 5.2 software for analysis [37].

### Statistical analysis

In this meta-analysis, the prognostic value of tissue VEGF expression in patients with ovarian cancer was measured by estimating the HR between high/positive expression of tissue VEGF groups and low/negative expression of tissue VEGF groups. The associated 95% CI were also measured. The heterogeneity between studies was evaluated with *P* value and *I*^*2*^. *I*^*2*^≥50% or *P*≤0.10 was deemed to existing significant heterogeneity [38, 39], and pooled HR was estimated using a random-effect model. On the contrary, if statistical study heterogeneity was not observed (*I*^*2*^≤50% and *P*≥0.10), a fixed effects model was used. For the pooled HR estimating OS, we performed subgroup analysis by detection method of tissue VEGF expression (IHC or PCR) and quality score (15, 16–18, 19) of included studies. additionally, publication bias was assessed by Begg’s and Egger’s test. If the shape of funnel plots revealed no obvious evidence of asymmetry, we considered that there was no obvious publication bias. All statistical analyses were performed using standard statistical procedures provided in RevMan 5.2 [37].

## CONCLUSIONS

In conclusion, positive/high expression of tissue VEGF may have a close association with worse survival in patients with ovarian cancer considerating of the evident statistical significance. Elevated tissue VEGF expression may be used as a prognostic biomarker for early identification of poor prognosis.

## References

[1] Siegel RL, Miller KD, Jemal A (2015). Cancer statistics, 2015. CA Cancer J Clin.

[2] Ferrara N (1995). The role of vascular endothelial growth factor in pathological angiogenesis. Breast Cancer Res Treat.

[3] Ferrara N (1999). Vascular endothelial growth factor: molecular and biological aspects. Curr Top Microbiol Immunol.

[4] Tempfer C, Obermair A, Hefler L, Haeusler G, Gitsch G, Kainz C (1998). Vascular endothelial growth factor serum concentrations in ovarian cancer. Obstet Gynecol.

[5] Tan XJ, Lang JH, Shen K, Wang L, Wu M, Xu XY (2008). [Correlation of preoperative serum vascular endothelial growth factor level with CA125 level in patients with epithelial ovarian cancer and its prognostic value] [Article in Chinese]. Zhonghua Fu Chan Ke Za Zhi.

[6] Bozas G, Terpos E, Gika D, Karadimou A, Dimopoulos MA, Bamias A (2010). Prechemotherapy serum levels of CD105, transforming growth factor beta2, and vascular endothelial growth factor are associated with prognosis in patients with advanced epithelial ovarian cancer treated with cytoreductivesurgery andplatinum-based chemotherapy. Int J Gynecol Cancer.

[7] Chen CA, Cheng WF, Lee CN, Chen TM, Kung CC, Hsieh FJ, Hsieh CY (1999). Serum vascular endothelial growth factor in epithelial ovarian neoplasms: correlation with patient survival. Gynecol Oncol.

[8] Brustmann H (2004). Vascular endothelial growth factor expression in serous ovarian carcinoma: relationship with topoisomerase II alpha and prognosis. Gynecol Oncol.

[9] Gadducci A, Viacava P, Cosio S, Cecchetti D, Fanelli G, Fanucchi A, Teti G, Genazzani AR (2003). Vascular endothelial growth factor (VEGF) expression in primary tumors and peritoneal metastases from patients with advanced ovarian carcinoma. Anticancer Res.

[10] Garzetti GG, Ciavattini A, Lucarini G, Pugnaloni A, De Nictolis M, Amati S, Romanini C, Biagini G (1999). Expression of vascular endothelial growth factor related to 72-kilodalton metalloproteinase immunostaining in patients with serous ovarian tumors. Cancer.

[11] Goodheart MJ, Ritchie JM, Rose SL, Fruehauf JP, De Young BR, Buller RE (2005). The relationship of molecular markers of p53 function and angiogenesis to prognosis of stage I epithelial ovarian cancer. Clin Cancer Res.

[12] Hartenbach EM, Olson TA, Goswitz JJ, Mohanraj D, Twiggs LB, Carson LF, Ramakrishnan S (1997). Vascular endothelial growth factor (VEGF) expression and survival in human epithelial ovarian carcinomas. Cancer Lett.

[13] Hata K, Nakayama K, Fujiwaki R, Katabuchi H, Okamura H, Miyazaki K (2004). Expression of the angopoietin-1, angopoietin-2, Tie2, and vascular endothelial growth factor gene in epithelial ovarian cancer. Gynecol Oncol.

[14] Ino K, Shibata K, Kajiyama H, Yamamoto E, Nagasaka T, Nawa A, Nomura S, Kikkawa F (2006). Angiotensin II type 1 receptor expression in ovarian cancer and its correlation with tumour angiogenesis and patient survival. Br J Cancer.

[15] Kassim SK, El-Salahy EM, Fayed ST, Helal SA, Helal T, Azzam Eel-D, Khalifa A (2004). Vascular endothelial growth factor and interleukin-8 are associated with poor prognosis in epithelial ovarian cancer patients. Clin Biochem.

[16] Li L, Liu B, Li X, Yang S, Xiao J, Chen M, Zhang Y, Ma J (2009). Vascular endothelial growth factor D and intratumoral lymphatics as independent prognostic factors in epithelial ovarian carcinoma. Anat Rec (Hoboken).

[17] Nakayama K, Kanzaki A, Hata K, Katabuchi H, Okamura H, Miyazaki K, Fukumoto M, Takebayashi Y (2002). Hypoxia-inducible factor 1 alpha (HIF-1 alpha) gene expression in human ovarian carcinoma. Cancer Lett.

[18] Nishida N, Yano H, Komai K, Nishida T, Kamura T, Kojiro M (2004). Vascular endothelial growth factor C and vascular endothelial growth factor receptor 2 are related closely to the prognosis of patients with ovarian carcinoma. Cancer.

[19] Raspollini MR, Amunni G, Villanucci A, Baroni G, Boddi V, Taddei GL (2004). Prognostic significance of microvessel density and vascular endothelial growth factor expression in advanced ovarian serous carcinoma. Int J Gynecol Cancer.

[20] Secord AA, Darcy KM, Hutson A, Lee PS, Havrilesky LJ, Grace LA, Berchuck A, Gynecologic Oncology Group study (2007). Co-expression of angiogenic markers and associations with prognosis in advanced epithelial ovarian cancer: a Gynecologic Oncology Group study. Gynecol Oncol.

[21] Shen GH, Ghazizadeh M, Kawanami O, Shimizu H, Jin E, Araki T, Sugisaki Y (2000). Prognostic significance of vascular endothelial growth factor expression in human ovarian carcinoma. Br J Cancer.

[22] Shimogai R, Kigawa J, Itamochi H, Iba T, Kanamori Y, Oishi T, Shimada M, Sato S, Kawaguchi W, Sato S, Terakawa N (2008). Expression of hypoxia-inducible factor 1alpha gene affects the outcome in patients with ovarian cancer. Int J Gynecol Cancer.

[23] Sinn BV, Darb-Esfahani S, Wirtz RM, Faggad A, Weichert W, Buckendahl AC, Noske A, Müller BM, Budczies J, Sehouli J, Braicu EI, Dietel M, Denkert C (2009). Vascular endothelial growth factor C mRNA expression is a prognostic factor in epithelial ovarian cancer as detected by kinetic RT-PCR in formalin-fixed paraffin-embedded tissue. Virchows Arch.

[24] Sundar SS, Zhang H, Brown P, Manek S, Han C, Kaur K, Charnock MF, Jackson D, Ganesan TS (2006). Role of lymphangiogenesis in epithelial ovarian cancer. Br J Cancer.

[25] Ueda M, Hung YC, Terai Y, Kanda K, Kanemura M, Futakuchi H, Yamaguchi H, Akise D, Yasuda M, Ueki M (2005). Vascular endothelial growth factor-C expression and invasive phenotype in ovarian carcinomas. Clin Cancer Res.

[26] Ferrara N, Davis-Smyth T (1997). The biology of vascular endothelial growth factor. Endocr Rev.

[27] Boocock CA, Charnock-Jones DS, Sharkey AM, McLaren J, Barker PJ, Wright KA, Twentyman PR, Smith SK (1995). Expression of vascular endothelial growth factor and its receptors flt and KDR in ovarian carcinoma. J Natl Cancer Inst.

[28] Mu J, Abe Y, Tsutsui T, Yamamoto N, Tai XG, Niwa O, Tsujimura T, Sato B, Terano H, Fujiwara H, Hamaoka T (1996). Inhibition of growth and metastasis of ovarian carcinoma by administering a drug capable of interfering with vascular endothelial growth factor activity. Jpn J Cancer Res.

[29] Brustmann H, Naude S (2002). Vascular endothelial growth factor expression in serous ovarian carcinoma: relationship with high mitotic activity and high FIGO stage. Gynecol Oncol.

[30] Mattern J, Stammler G, Koomagi R, Wallwiener D, Kaufmann M, Volm M (1997). Association of vascular endothelial growth factor expression with tumor cell proliferation in ovarian carcinoma. Anticancer Res.

[31] Niki T, Iba S, Tokunou M, Yamada T, Matsuno Y, Hirohashi S (2000). Expression of vascular endothelial growth factors A, B, C, and D and their relationships to lymph node status in lung adenocarcinoma. Clin Cancer Res.

[32] Poesen K, Lambrechts D, Van Damme P, Dhondt J, Bender F, Frank N, Bogaert E, Claes B, Heylen L, Verheyen A, Raes K, Tjwa M, Eriksson U (2008). Novel role for vascular endothelial growth factor (VEGF) receptor-1 and its ligand VEGF-B in motor neuron degeneration. J Neurosci.

[33] Räsänen M, Degerman J, Nissinen TA, Miinalainen I, Kerkelä R, Siltanen A, Backman JT, Mervaala E, Hulmi JJ, Kivelä R, Alitalo K (2016). VEGF-B gene therapy inhibits doxorubicin-induced cardiotoxicity by endothelial protection. Proc Natl Acad Sci U S A.

[34] Salven P, Lymboussaki A, Heikkilä P, Jääskela-Saari H, Enholm B, Aase K, von Euler G, Eriksson U, Alitalo K, Joensuu H (1998). Vascular endothelial growth factors VEGF-B and VEGF-C are expressed in human tumors. Am J Pathol.

[35] Woolard J, Bevan HS, Harper SJ, Bates DO (2009). Molecular diversity of VEGF-A as a regulator of its biological activity. Microcirculation.

[36] McShane LM, Altman DG, Sauerbrei W, Taube SE, Gion M, Clark GM (2006). REporting recommendations for tumor MARKer prognostic studies (REMARK). Breast Cancer Res Treat.

[37] Review Manager (RevMan) [Computer Program]. Version 5.2. Copenhagen: The Nordic Cochrane Centre, The Cochrane Collaboration, 2012

[38] Higgins JPT, Green S Cochrane Handbook for Systematic Rev iews of Interventions.

[39] University of York Centre for Reviews and Dissemination (2009). Systematic Reviews: CRD’s Guidance for Undertaking Reviews in Health Care.

